# Stabilizing Water-in-Water Emulsions Using Oil Droplets

**DOI:** 10.3390/molecules30153120

**Published:** 2025-07-25

**Authors:** Jean-Paul Douliez, Laure Béven

**Affiliations:** Biologie du Fruit et Pathologie, INRAE, Univ. Bordeaux, UMR 1332, F-33140 Villenave d’Ornon, France; laure.beven@inrae.fr

**Keywords:** water-in-water emulsion, stabilization, double emulsion

## Abstract

The production of water-in-water emulsion droplets, the coalescence of which is prevented by adding oil-in-water micrometric droplets, is reported. Hexadecane (O) and cetyl trimethyl ammonium bromide (CTAB) were added to a W/W emulsion made of dextran (Dex)-enriched droplets in a Polyethyleglycol (PEG)-enriched continuous phase, and the mixture was further sonicated. Using Nile red to label the oil droplets enabled the observation of their presence at the surface of Dex droplets (5 µm), allowing for stabilizing them, preventing coalescence of the W/W emulsion, and mimicking W/O/W double emulsions. The addition of sulfate derivative of Dextran (DexSulf) allowed for stable droplets of a slightly larger diameter. By contrast, the addition of carboxymethyl Dextran (CMDex) destabilized the initial aqueous double-like emulsion, yielding sequestration of the oil droplets within the Dex-rich phase. Interestingly, addition of DexSulf to that unstable emulsion re-yielded stable droplets. Similar findings (destabilization) were obtained when adding sodium dodecyl sulfate (SDS) to the initial double-like emulsion, which reformed stable droplets when adding positively charged Dextran (DEAEDex) derivatives. The use of fluorescently (FITC) labeled derivatives of Dextran (Dex, CMDex, DEAEDex, and DexSulf) allowed us to follow their position within, out of, or at the interface of droplets in the above-mentioned mixtures. These findings are expected to be of interest in the field of materials chemistry.

## 1. Introduction

Double water-in-oil-in-water emulsions (W/O/W) are attractive for technological applications, owing to their ability to gather apolar and polar phases in a single compartment, then affording the encapsulation of both hydrophilic and hydrophobic cargoes. They were initially produced via a two-step method [1] that consists of preparing W/O droplets that are further emulsified with water. This requires the use of hydrophilic and lipophilic emulsifiers that span both interfaces and a relatively high amount of the last one to prevent release of the internal aqueous droplet during the second emulsification step [2,3,4,5,6,7,8,9,10,11,12,13,14,15]. This method allows for the facile encapsulation of hydrophilic cargos to be dispersed in the initial W/O droplets.

An alternative method was developed recently, affording one-step preparation of such emulsions by using blends of surfactants or amphiphilic polymers [5,11,16,17,18,19,20,21,22,23,24,25,26,27]. This way is more attractive than the two-step method, owing to its simplicity. However, because they are obtained by simply mixing oil and water (and emulsifiers), the aqueous inner droplet of the double emulsion has the same composition as that of the continuous phase so that the encapsulation of cargos is much less efficient, mostly remaining in the outer phase. Another direction is a spontaneous emulsification that still proceeds in one step and is achieved by osmose-induced diffusion of water in oil droplets. This was first observed when forming O/W droplets stabilized by amphiphilic polymers that also contained salts, dispersed in oil, which contributed to the formation of small aqueous droplets within oil [17,18,22,23,24,25,26,27,28,29,30,31,32,33,34].

Another alternative method under study would be to produce double emulsions starting from water-in-water emulsions. These are made of mixtures of, for instance, two segregative polymers, e.g., polyethylene glycol (PEG) and Dextran (Dex), that phase separate at a given concentration in water [35,36,37,38,39,40,41]. Depending on the concentration and the molecular weight of both polymers, one can form Dex-rich droplets in a continuous PEG-rich continuous phase. Such systems are also known as aqueous two-phase systems (ATPSs). These all-aqueous emulsions can also form from aggregative polymers, e.g., oppositely charged polymers, also including small polyampholytes such as adenosine triphosphate [42,43,44,45,46,47,48]. In this case, both polymers are gathered within aqueous droplets, with some remaining in the continuous phase. Such systems are also known as (complex or single) coacervates in the literature. When formed, these aqueous droplets are not stable, and generally, coalescence occurs, yielding macroscopic phase separation. A strong effort has been devoted to the stabilization of these droplets in the last few years [14,36,49,50,51,52,53,54,55,56,57,58,59,60,61,62,63,64,65,66,67,68,69,70,71,72]. Such droplets can spontaneously sequester cargos, and this represents a strong advantage for preparing synthetic cells [73] or for building capsules that could encapsulate chemicals for various applications [36].

**Scheme 1:**
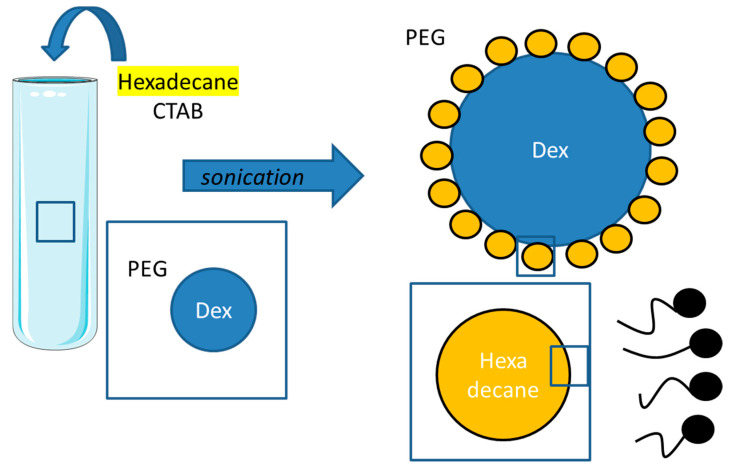
Schematic representation of the procedure to form double-like or Pickering emulsion. Oil (hexadecane, yellow) and surfactant (CTAB, dark) are mixed with a pre-prepared W/W made of Dex-rich droplets (blue) embedded in a PEG-rich continuous phase. The sample is then sonicated to produce Dex-rich droplets, on which small, micrometric oil-in-water droplets become spontaneously adsorbed.

Using the alternative above-mentioned method to produce double emulsion forms all-aqueous emulsions would allow for the sequestration of cargos within the W/W droplets and then their strict encapsulation in the W/O/W emulsion. Here, aqueous two-phase systems are stabilized by small oil-in-water emulsion droplets, which come at the surface of Dex-rich droplets, forming a Pickering-like aqueous emulsion or a W/O/W double-like emulsion (see Scheme 1). Adding oppositely charged surfactants or polymers allowed for the destabilization and/or reinforced stabilization of the initial emulsion.

## 2. Results and Discussion

The W/W all-aqueous emulsion was made of dextran (Dex, 500 kDa, 32.5 mg/mL) and polyethylene glycol (PEG, 20 kDa, 70 mg/mL) dispersed in water. At these concentrations of polymers, and after vigorous stirring, 5–50 µm dextran-enriched droplets in a PEG continuous phase were produced, forming a slightly turbid dispersion. If left at rest, Dex-rich droplets coalesced, further yielding macroscopic phase separation in about 1 h, a well-known behavior [35,36].

**Figure 1:**
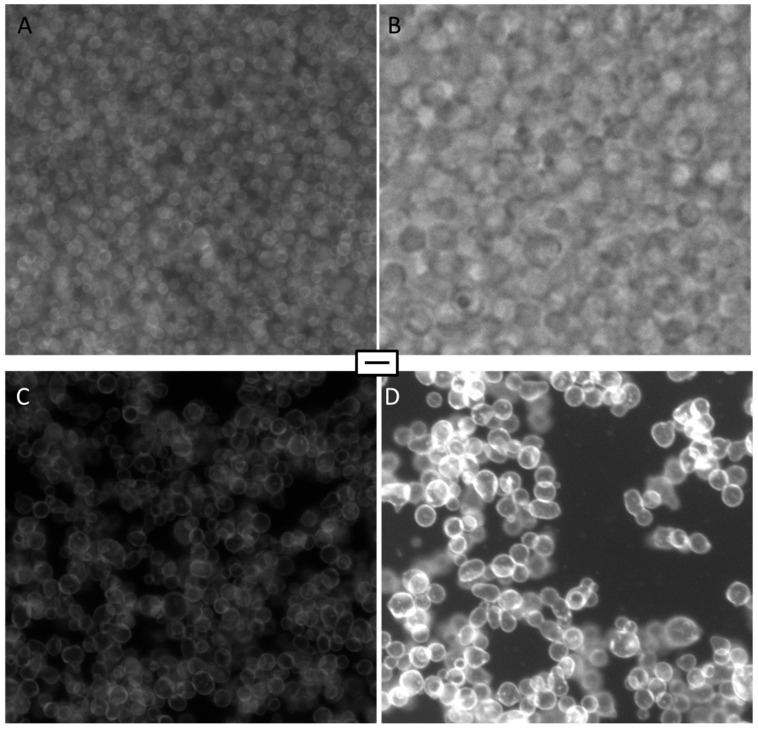
Microscopy images (epifluorescence in false color: (**A**,**C**,**D**) (scale bar 10 μm); bright field: (**B**) (scale bar 5 μm) of double-like emulsions.) (**A**), red channel. Emulsion produced with Nile red added with hexadecane. (**B**), same emulsion. The size of the image has been increased twice. (**C**,**D**), red and green channel. Same emulsion as in the case ‘A’ but after addition of DexSulfFITC.

To this aqueous emulsion (5 mL), hexadecane (200 µL, in which some Nile red was also added) and CTAB dispersed in water (5%, 100 µL) were added, and the mixture was further sonicated for 30 s. This yielded a very turbid, milky solution formed by small dextran-rich droplets surrounded by a red fluorescent shell with a relatively monodisperse diameter lower than 5 µm (Figure 1A,B). These small droplets no longer coalesced as observed in the oil and CTAB-free all-aqueous emulsion. The use of Nile red allowed for labeling the oil hexadecane phase, and the observation of small circles by epifluorescence (Figure 1A) attested that the shell of these dextran-rich droplets is made of small oil-in-water droplets (stabilized by CTAB) that formed upon sonication of the mixture (note that here, the water phase is, in fact, a PEG-rich aqueous phase). We do not believe that a continuous layer of hexadecane formed at the surface of the dextran-rich droplets simply because of the sonication procedure, which rather allows for the formation of small micrometric oil droplets in these conditions. Obviously, the size of oil-in-water droplets, as produced via sonication, is expected to range from 100 nm to 1 µm so that these are not distinctly visible on microscopy images. The solution was stable for months and showed creaming but no coalescence of Dex-rich droplets.

Clearly, a Pickering-like W/W emulsion formed in these conditions, with oil droplets playing the role of particles. In other words, these compartments also resemble W/O/W double emulsion, although the Dex-rich droplets are not covered by a regular oil shell but by juxtaposed small droplets (see also Scheme 1). Similar findings have been reported by others when using lipid particles that also spread at the Dex-rich interface with PEG [14].

An alternative method was also used to produce such a double-like emulsion. Instead of adding oil and CTAB in the pre-formed all-aqueous emulsion, an oil-in-water emulsion was first prepared by adding oil and CTAB in a more concentrated ***single*** PEG aqueous phase (20 kDa, 8%, 4 mL + 200 µL Hexadecane and 100 µL CTAB (5%)). This mixture was sonicated as above to form an oil-in-water (PEG) emulsion. Then, a concentrated dispersion of Dex (21%, 1 mL) was added upon vigorous agitation, affording the double-like or Pickering emulsion as described above, with droplets having the same diameter.

The stability and robustness of such double-like emulsions were further evaluated by adding other chemicals to the double-like emulsion prepared as described above (1 mL). The sulfate derivative of Dextran (DexSulf, 500 kDa, 5% in water, 200 µL) was added upon vigorous agitation. This yielded droplets having a slightly higher diameter of about 8 µm, and noticeably, the shape of these compartments was also less spherical (Figure 1C,D). Again, the use of Nile red allowed for observing the small oil-in-water droplets shell at the surface of the Dex-rich droplets (Figure 1C). In the same way, the use of FITC-labeled DexSulf showed fluorescent circles, attesting that this polymer was also localized at the interface (Figure 1D), which very logically interacts with positively charged oil-in-water droplets stabilized by CTAB. Again, these dispersions were stable for months without noticeable observation of coalescence events in the double-like emulsion, since the size and even the shape of droplets were the same after such a long period of time. It is expected that the DexSulf coupled to the oil-in-water droplets stabilized by CTAB forms a robust shell around the Dex-rich droplets.

**Figure 2:**
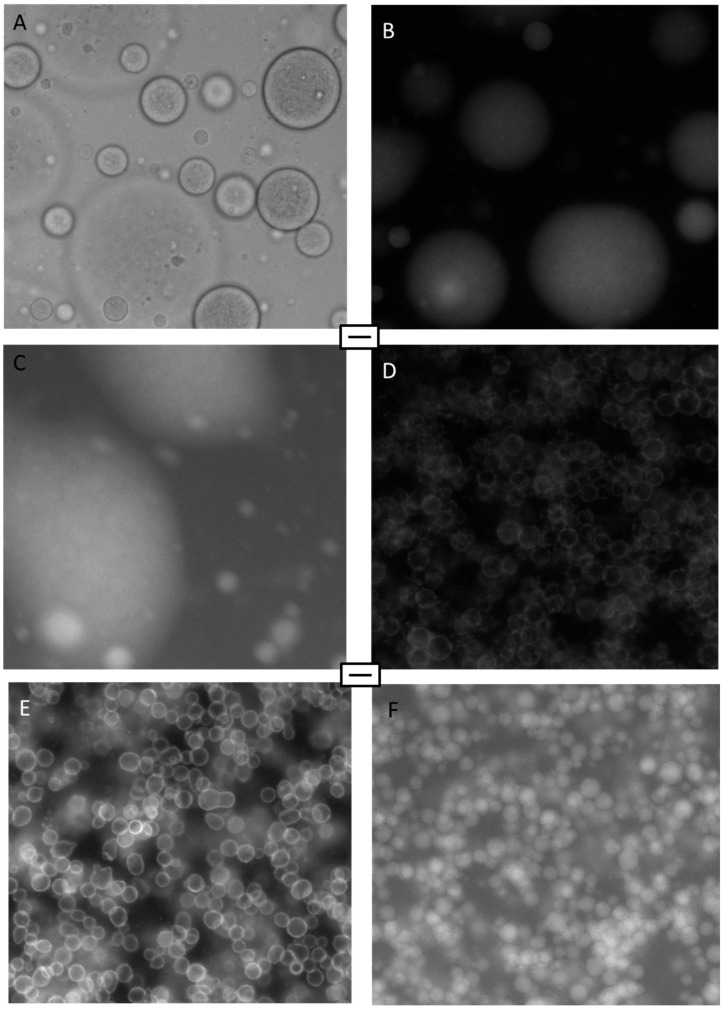
Microscopy images (epifluorescence, except (**A**): bright field); scale bar is the same for all images and stands for 10 µm. (**A**–**C**): Double-like emulsion (produced with Nile red in the hexadecane) in the presence of CM-DexFITC in the red and green channel (image (**B**,**C**), respectively). (**D**,**E**); Same emulsion but in the presence of DexSulf, (**D**) in the red channel, (**E**) in the green channel (here, DexSulfFITC was used, and CM-Dex was not fluorescently labeled), and (**F**) in the green channel (here, CM-DexFITC was used, and DexSulf was not fluorescently labeled).

By contrast, the addition of a carboxymethyl Dextran derivative (CM-Dex, 150 kDa, 5% in water, 200 µL in 1 mL double-like emulsion) to the initial double-like emulsion yielded destabilization (Figure 2A–C). Droplets exhibited a size similar to that of the ‘simple’ Dex-in-PEG all-aqueous emulsion. The coalescence of droplets was also observed, which could also spread on the glass microscopy slide. The use of Nile red and CM-DexFITC allowed for observing red and green patches and droplets (Figure 2B,C). In contrast to what occurs when adding DexSulf, in this case, this suggests that CM-Dex probably covered the small oil-in-water droplets, interacting with CTAB and then becoming sequestered within the Dex-rich phase. This could be expected since CM-Dex alone is also sequestered within the Dex-rich phase in the ‘simple’ Dex-in-PEG all-aqueous emulsion. The all-aqueous emulsion was no longer stabilized in these conditions.

Then, to this unstable emulsion, DexSulf was added as above under vigorous vortexing (DexSulf, 200 µL in 1 mL double-like emulsion that also contained 200 µL CM-Dex). This yielded stable and smaller droplets, the sizes of which were similar to those observed in Figure 1. The use of both Nile red and DexSulfFITC allowed for observing fluorescent circles (Figure 2D,E), attesting that oil droplets interacted with DexSulf and again covered Dex-rich droplets. By contrast, the use of CM-DexFITC allowed observing small fluorescent droplets (Figure 2F), showing that this polymer remained entrapped within the Dex-rich phase. It is expected that the sulfate groups of DexSulf *interact much more strongly* with the tetrabutyl ammonium moiety of CTAB than do the carboxylate groups of CM-Dex. As a consequence, the oil droplets stabilized by CTAB are no longer covered by CM-Dex but by DexSulf, allowing these colloidal droplets to move back to the interface of Dex-rich droplets and the PEG-rich continuous phase, leaving CM-Dex within droplets. This can then be a nice way to encapsulate negatively charged chemicals in such double-like emulsion.

**Figure 3:**
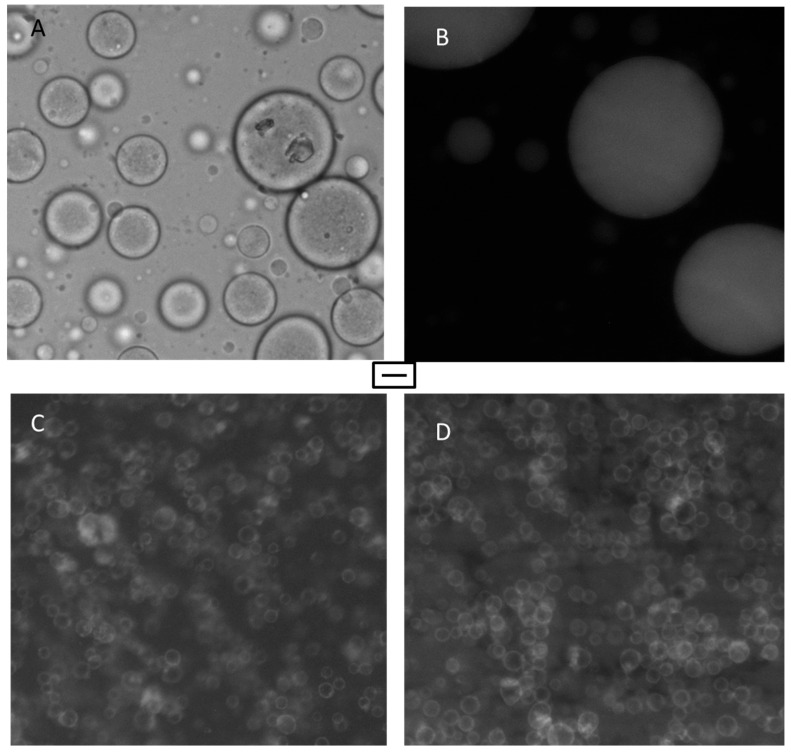
Microscopy images: (**A**) bright field and (**B**–**D**) epifluorescence in false color; the scale bar is the same for all images and stands for 10 µm. (**A**,**B**) Red channel, for a double-like emulsion destabilized by using SDS. (**C**) Red channel and (**D**) green channel, with the same emulsion as above, after addition of DEAE-DexFITC.

Finally, the robustness of the double-like emulsion was also evaluated upon addition of the negatively charged surfactant, sodium dodecyl sulfate (SDS, 5% in water, 50 µL in 1 mL double-like emulsion). SDS is expected to interact with the CTAB used to stabilize the oil-in-water droplets. This negatively charged surfactant induced the destabilization of the double-like emulsion, forming Dex-rich droplets with larger sizes (Figure 3A,B), which could also coalesce with time. This behavior resembles that observed previously when adding CM-Dex. It is believed that SDS micelles covered the oil droplets (initially covered by CTAB), which were then sequestered within the Dex-rich phase, as occurred previously when adding CM-Dex. Here, the addition of DexSulf again induced a transition to stable smaller droplets, as observed in the latter case (Figure 2). In the same way, instead of DexSulf, the addition of positively charged Dex derivative DEAE-Dex also induced a transition to stable smaller droplets (Figure 3C,D), which no longer coalesced. That polymer is expected to recover the oil-in-water droplets that were initially stabilized by CTAB but were also covered with SDS micelles, allowing for these oil droplets to come back to the Dex-rich droplet interface, yielding their stabilization. This feature was again clearly observed by epifluorescence microscopy using DEAE-DexFITC and Nile red.

In summary, small oil-in-water droplets produced ‘in situ’ via sonication in the W/W emulsion allowed for the stabilization of the latter. This yields the formation of a Pickering or double-like emulsion with oil droplets juxtaposed at the Dex-rich droplet interface with the PEG-rich continuous phase. Using additional surfactants or polymers allowed for the destabilization of the double-like emulsion, which could reform stable droplets upon addition of another polymer. The very long-term stability of such emulsions was not studied in this work because the formation of such Pickering emulsions, already stable enough for weeks or months, could allow for preparing capsules by adding chemical cross-linkers, and we are investigating this avenue in the future. This work shows an additional way to stabilize all-aqueous emulsions and could find applications in the domain of materials chemistry and encapsulation.

## 3. Materials and Methods

An 80 mL stock solution of the W/W all-aqueous emulsion was prepared using dextran (Dex, 500 kDa, 32.5 mg/mL, Pharmacia T500, Sigma-Aldrich, St. Quentin, France) and polyethylene glycol (PEG, 20 kDa, 70 mg/mL, Sigma-Aldrich, St. Quentin, France) dispersed in water. In total, 5 mL of this emulsion was further poured into different tubes. Then, hexadecane and CTAB (initially dispersed in water at a concentration of 5% W/W), both from Sigma-Aldrich, were added, and mixtures were subjected to ultrasonication with a VibraCell SonicMaterials Inc., Danbury, CT, USA, power 4, for 30 s.

All FITC-labeled Dextran derivatives were from Sigma-Aldrich and were prepared at a concentration of 5% in water. Unlabeled Dextran derivatives and SDSs were also from Sigma-Aldrich and prepared at a concentration of 5% W/W in water. Different volumes of these chemicals were added, as described in the main text upon vigorous agitation.

The references of those chemicals are as follows (MW when available and ref from Sigma): Dextran sulfate (500 kDa, ref D-8906), DexSulfFITC (500 kDa, ref 51923), carboxymethyl Dextran (CM-Dex ref 86524), CM-DexFITC (150 kDa, ref 74817), Dextran FITC (DexFITC, 500 kDa, ref 46947), DEAE-Dex (ref D9885), and DEAE-DexFITC (150 kDa, ref 75005).

Optical microscopy imaging was performed on a Leica DMI 4000B (Leica, Paris, France), inverted epifluorescence microscope equipped with a ×40 lens and a CoolLED light source combined with appropriate filter cubes to select the excited and emitted fluorescence wavelength range. Images were acquired using MicroManager and processed with ImageJ.

## Data Availability

Data are contained within the article.

## References

[1] Matsumoto S., Kita Y., Yonezawa D. (1976). An attempt at preparing water-in-oil-in-water multiple-phase emulsions. J. Colloid Interface Sci..

[2] Ficheux M.F., Bonakdar L., Leal-Calderon F., Bibette J. (1998). Some Stability Criteria for Double Emulsions. Langmuir.

[3] Kanouni M., Rosano H.L., Naouli N. (2002). Preparation of a stable double emulsion (W1/O/W2): Role of the interfacial films on the stability of the system. Adv. Colloid Interface Sci..

[4] Ding S., Anton N., Akram S., Er-Rafik M., Anton H., Klymchenko A., Yu W., Vandamme T.F., Serra C.A. (2017). A new method for the formulation of double nanoemulsions. Soft Matter.

[5] Hanson J.A., Chang C.B., Graves S.M., Li Z., Mason T.G., Deming T.J. (2008). Nanoscale double emulsions stabilized by single-component block copolypeptides. Nature.

[6] Utada A.S., Lorenceau E., Link D.R., Kaplan P.D., Stone H.A., Weitz D.A. (2005). Monodisperse Double Emulsions Generated from a Microcapillary Device. Science.

[7] Wang W., Zhang M.-J., Chu L.-Y. (2014). Microfluidic approach for encapsulation via double emulsions. Curr. Opin. Pharmacol..

[8] Garti N., Bisperink C. (1998). Double emulsions: Progress and applications. Curr. Opin. Colloid Interface Sci..

[9] Garti N., Aserin A. (1996). Double emulsions stabilized by macromolecular surfactants. Adv. Colloid Interface Sci..

[10] Muschiolik G., Dickinson E. (2017). Double Emulsions Relevant to Food Systems: Preparation, Stability, and Applications. Compr. Rev. Food Sci. Food Saf..

[11] Ding S., Serra C.A., Vandamme T.F., Yu W., Anton N. (2019). Double emulsions prepared by two-step emulsification: History, state-of-the-art and perspective. J. Control. Release.

[12] Garti N. (1997). Double emulsions scope, limitations and new achievements. Colloids Surf. A.

[13] Florence A.T., Whitehill D. (1981). Some features of breakdown in water-in-oil-in-water multiple emulsions. J. Colloid Interface Sci..

[14] Dumas F., Benoit J.-P., Saulnier P., Roger E. (2021). A new method to prepare microparticles based on an Aqueous Two-Phase system (ATPS), without organic solvents. J. Colloid Interface Sci..

[15] Florence A.T., Whitehill D. (1982). The formulation and stability of multiple emulsions. Int. J. Pharm..

[16] Gao H., Ma L., Cheng C., Liu J., Liang R., Zou L., Liu W., McClements D.J. (2021). Review of recent advances in the preparation, properties, and applications of high internal phase emulsions. Trends Food Sci. Technol..

[17] Hong L., Sun G., Cai J., Ngai T. (2012). One-Step Formation of W/O/W Multiple Emulsions Stabilized by Single Amphiphilic Block Copolymers. Langmuir.

[18] Zhang T., Ngai T. (2021). One-Step Formation of Double Emulsions Stabilized by PNIPAM-based Microgels: The Role of Co-monomer. Langmuir.

[19] Wang Z., Song J., Zhang S., Xu X.-Q., Wang Y. (2017). Formulating Polyethylene Glycol as Supramolecular Emulsifiers for One-Step Double Emulsions. Langmuir.

[20] Kim S., Kim K., Choi S.Q. (2018). Controllable one-step double emulsion formation via phase inversion. Soft Matter.

[21] Protat M., Bodin N., Gobeaux F., Malloggi F., Daillant J., Pantoustier N., Guenoun P., Perrin P. (2016). Biocompatible Stimuli-Responsive W/O/W Multiple Emulsions Prepared by One-Step Mixing with a Single Diblock Copolymer Emulsifier. Langmuir.

[22] Li R., Wang Z., Tao X., Jia J., Lian X., Wang Y. (2016). Redox-Driven Spontaneous Double Emulsion. ACS Macro Lett..

[23] Qian Q., Huang X., Zhang X., Xie Z., Wang Y. (2013). One-step Preparation of Macroporous Polymer Particles with Multiple Interconnected Chambers: A Candidate for Trapping Biomacromolecules. Angew. Chem. Int. Ed..

[24] Wang D., Huang X., Wang Y. (2014). Managing the Phase Separation in Double Emulsion by Tuning Amphiphilicity via a Supramolecular Route. Langmuir.

[25] Huang X., Qian Q., Wang Y. (2014). Anisotropic Particles from a One-Pot Double Emulsion Induced by Partial Wetting and Their Triggered Release. Small.

[26] Jiang H., Sheng Y., Ngai T. (2020). Pickering emulsions: Versatility of colloidal particles and recent applications. Curr. Opin. Colloid Interface Sci..

[27] Ao Z., Yang Z., Wang J., Zhang G., Ngai T. (2009). Emulsion-Templated Liquid Core-Polymer Shell Microcapsule Formation. Langmuir.

[28] Huang X., Fang R., Wang D., Wang J., Xu H., Wang Y., Zhang X. (2014). Tuning Polymeric Amphiphilicity via Se-N Interactions: Towards One-Step Double Emulsion for Highly Selective Enzyme Mimics. Small.

[29] Wang Z., Wang Y. (2016). Tuning Amphiphilicity of Particles for Controllable Pickering Emulsion. Materials.

[30] Chen Y., Wang Z., Wang D., Ma N., Li C., Wang Y. (2016). Surfactant-Free Emulsions with Erasable Triggered Phase Inversions. Langmuir.

[31] Sun G., Liu M., Zhou X., Hong L., Ngai T. (2014). Influence of asymmetric ratio of amphiphilic diblock copolymers on one-step formation and stability of multiple emulsions. Colloids Surf. A.

[32] Bae J., Russell T.P., Hayward R.C. (2014). Osmotically Driven Formation of Double Emulsions Stabilized by Amphiphilic Block Copolymers. Angew. Chem. Int. Ed..

[33] Zhu J., Hayward R.C. (2012). Interfacial tension of evaporating emulsion droplets containing amphiphilic block copolymers: Effects of solvent and polymer composition. J. Colloid Interface Sci..

[34] Zhu J., Hayward R.C. (2008). Hierarchically Structured Microparticles Formed by Interfacial Instabilities of Emulsion Droplets Containing Amphiphilic Block Copolymers. Angew. Chem. Int. Ed..

[35] Esquena J. (2016). Water-in-water (W/W) emulsions. Curr. Opin. Colloid Interface Sci..

[36] Perro A., Coudon N.M., Chapel J.-P., Martin N., Béven L., Douliez J.-P. (2022). Building micro-capsules using water-in-water emulsion droplets as templates. J. Colloid Interface Sci..

[37] Torre P., Keating C.D., Mansy S.S. (2014). Multiphase Water-in-Oil Emulsion Droplets for Cell-Free Transcription-Translation. Langmuir.

[38] Dominak L.M., Gundermann E.L., Keating C.D. (2010). Microcompartmentation in Artificial Cells: pH-Induced Conformational Changes Alter Protein Localization. Langmuir.

[39] Aumiller W.M., Keating C.D. (2017). Experimental models for dynamic compartmentalization of biomolecules in liquid organelles: Reversible formation and partitioning in aqueous biphasic systems. Adv. Colloid Interface Sci..

[40] Helfrich M.R., Mangeney-Slavin L.K., Long M.S., Djoko K.Y., Keating C.D. (2002). Aqueous phase separation in giant vesicles. J. Am. Chem. Soc..

[41] Keating C.D. (2012). Aqueous Phase Separation as a Possible Route to Compartmentalization of Biological Molecules. Acc. Chem. Res..

[42] Vogele K., Frank T., Gasser L., Goetzfried M.A., Hackl M.W., Sieber S.A., Simmel F.C., Pirzer T. (2018). Towards synthetic cells using peptide-based reaction compartments. Nat. Commun..

[43] Williams David S., Patil Avinash J., Mann S. (2014). Spontaneous Structuration in Coacervate-Based Protocells by Polyoxometalate-Mediated Membrane Assembly. Small.

[44] Moreau N.G., Martin N., Gobbo P., Tang T.Y.D., Mann S. (2020). Spontaneous membrane-less multi-compartmentalization via aqueous two-phase separation in complex coacervate micro-droplets. Chem. Commun..

[45] Sakuta H., Fujita F., Hamada T., Hayashi M., Takiguchi K., Tsumoto K., Yoshikawa K. (2020). Self-Emergent Protocells Generated in an Aqueous Solution with Binary Macromolecules through Liquid-Liquid Phase Separation. ChemBioChem.

[46] Koga S., Williams D.S., Perriman A.W., Mann S. (2011). Peptide-nucleotide microdroplets as a step towards a membrane-free protocell model. Nat. Chem..

[47] Martin N. (2019). Dynamic Synthetic Cells Based on Liquid-Liquid Phase Separation. ChemBioChem.

[48] Martin N., Douliez J.-P., Qiao Y., Booth R., Li M., Mann S. (2018). Antagonistic chemical coupling in self-reconfigurable host-guest protocells. Nat. Commun..

[49] Poortinga A.T. (2008). Microcapsules from Self-Assembled Colloidal Particles Using Aqueous Phase-Separated Polymer Solutions. Langmuir.

[50] Douliez J.-P., Perro A., Béven l. (2019). Stabilization of all-in-water emulsions to form capsules as artificial cells. ChemBioChem.

[51] Ben Ayed E., Cochereau R., Dechancé C., Capron I., Nicolai T., Benyahia L. (2018). Water-In-Water Emulsion Gels Stabilized by Cellulose Nanocrystals. Langmuir.

[52] Merland T.O., Waldmann L.A., Guignard O., Tatry M.-C., Wirotius A.-L., Lapeyre V., Garrigue P., Nicolai T., Benyahia L., Ravaine V. (2022). Thermo-induced inversion of water-in-water emulsion stability by bis-hydrophilic microgels. J. Colloid Interface Sci..

[53] de Freitas R.A., Nicolai T., Chassenieux C., Benyahia L. (2016). Stabilization of Water-in-Water Emulsions by Polysaccharide-Coated Protein Particles. Langmuir.

[54] Nguyen B.T., Wang W., Saunders B.R., Benyahia L., Nicolai T. (2015). pH-Responsive Water-in-Water Pickering Emulsions. Langmuir.

[55] Balakrishnan G., Nicolai T., Benyahia L., Durand D. (2012). Particles Trapped at the Droplet Interface in Water-in-Water Emulsions. Langmuir.

[56] Nicolai T., Murray B. (2017). Particle stabilized water in water emulsions. Food Hydrocoll..

[57] Gonzalez-Jordan A., Nicolai T., Benyahia L. (2016). Influence of the Protein Particle Morphology and Partitioning on the Behavior of Particle-Stabilized Water-in-Water Emulsions. Langmuir.

[58] Gonzalez-Jordan A., Nicolai T., Benyahia L. (2018). Enhancement of the particle stabilization of water-in-water emulsions by modulating the phase preference of the particles. J. Colloid Interface Sci..

[59] Machado J., Benyahia L., Nicolai T. (2021). Effect of adding a third polysaccharide on the adsorption of protein microgels at the interface of polysaccharide-based water in water emulsions. J. Colloid Interface Sci..

[60] Gonzalez-Jordan A., Benyahia L., Nicolai T. (2017). Cold gelation of water in water emulsions stabilized by protein particles. Colloids Surf. A.

[61] Zhu S., Forth J., Xie G., Chao Y., Tian J., Russell T.P., Shum H.C. (2020). Rapid Multilevel Compartmentalization of Stable All-Aqueous Blastosomes by Interfacial Aqueous-Phase Separation. ACS Nano.

[62] Dewey D.C., Strulson C.A., Cacace D.N., Bevilacqua P.C., Keating C.D. (2014). Bioreactor droplets from liposome-stabilized all-aqueous emulsions. Nat. Commun..

[63] Douliez J.-P., Perro A., Chapel J.-P., Goudeau B., Béven L. (2018). Preparation of Template-Free Robust Yolk-Shell Gelled Particles from Controllably Evolved All-in-Water Emulsions. Small.

[64] Douliez J.-P., Martin N., Beneyton T., Eloi J.-C., Chapel J.-P., Navailles L., Baret J.-C., Mann S., Béven L. (2018). Preparation of Swellable Hydrogel-Containing Colloidosomes from Aqueous Two-Phase Pickering Emulsion Droplets. Angew. Chem. Int. Ed..

[65] Ganley W.J., Ryan P.T., van Duijneveldt J.S. (2017). Stabilisation of water-in-water emulsions by montmorillonite platelets. J. Colloid Interface Sci..

[66] Bago Rodriguez A.M., Binks B.P., Sekine T. (2016). Novel stabilisation of emulsions by soft particles: Polyelectrolyte complexes. Faraday Discuss..

[67] Zhang S., Contini C., Hindley J.W., Bolognesi G., Elani Y., Ces O. (2021). Engineering motile aqueous phase-separated droplets via liposome stabilisation. Nat. Commun..

[68] Peddireddy K.R., Nicolai T., Benyahia L., Capron I. (2016). Stabilization of Water-in-Water Emulsions by Nanorods. ACS Macro Lett..

[69] Tea L., Nicolai T., Renou F. (2019). Stabilization of Water-In-Water Emulsions by Linear Homo-Polyelectrolytes. Langmuir.

[70] Nguyen B.T., Nicolai T., Benyahia L. (2013). Stabilization of Water-in-Water Emulsions by Addition of Protein Particles. Langmuir.

[71] Coudon N., Navailles L., Nallet F., Ly I., Bentaleb A., Chapel J.-P., Béven L., Douliez J.-P., Martin N. (2022). Stabilization of all-aqueous droplets by interfacial self-assembly of fatty acids bilayers. J. Colloid Interface Sci..

[72] Beldengré Y., Dallaris V., Jan C., Protat R., Miras J., Calvo M., Garca-Celma M.J., Esquena J. (2019). Formation and stabilization of multiple water-in-water-in-water (W/W/W) emulsions. Food Hydrocoll..

[73] Abbas M., Lipiski W.P., Wang J., Spruijt E. (2021). Peptide-based coacervates as biomimetic protocells. Chem. Soc. Rev..

